# Post-COVID-19 Small Fiber Neuropathy as a New Emerging Quality of Life-Threatening Disease: A Systematic Review

**DOI:** 10.3390/microorganisms13020328

**Published:** 2025-02-02

**Authors:** Francesca Bandinelli, Marco Di Carlo, Virginia Alba Colantuono, Filippo Nozzoli, Fausto Salaffi, Barbara Chiocchetti, Elena Nucci, Alessandra Mastricci, Eleonora Gherardi, Mirko Manetti

**Affiliations:** 1 Rheumatology Department, Santa Maria Nuova Hospital, Usl Tuscany Center, 50122 Florence, Italy; 2 Rheumatology Unit, Department of Clinical and Molecular Sciences, Università Politecnica of Marche, Carlo Urbani Hospital, Jesi, 60035 Ancona, Italy; dica.marco@yahoo.it (M.D.C.); fausto.salaffi@gmail.com (F.S.); 3 Section of Dermatology, Department of Health Sciences, University of Florence, 50125 Florence, Italy; virginiaalba.colantuono@unifi.it (V.A.C.); eleonora.gherardi@unifi.it (E.G.); 4 Section of Pathological Anatomy, Department of Health Sciences, University of Florence, 50139 Florence, Italy; filippo.nozzoli@unifi.it; 5 Neurology Department, San Giovanni di Dio Hospital, Usl Tuscany Center, 50143 Florence, Italy; barbara.chiocchetti@uslcentro.toscana.it; 6 Histopathology and Molecular Diagnostics, Careggi University Hospital, University of Florence, 50139 Florence, Italy; elena.nucci@unifi.it; 7 Section of Clinical Pharmacology and Oncology, Department of Health Sciences, University of Florence, 50139 Florence, Italy; alessandra.mastricci@unifi.it; 8 Section of Anatomy and Histology, Department of Experimental and Clinical Medicine, University of Florence, 50134 Florence, Italy; mirko.manetti@unifi.it

**Keywords:** small fiber neuropathy, COVID-19, post-acute sequelae of COVID-19, arthritis

## Abstract

Post-acute sequelae of COVID-19 (PASC) syndrome is considered an emergent and diffuse multidisciplinary problem. Compelling evidence suggests that COVID-19 increases symptoms of pre-existent small fiber neuropathy (SFN) and might trigger de novo onset of SFN. In this systematic review, for the first time, we provide a comprehensive overview of the clinical and diagnostic features of PASC-SFN, including the accompanying disorders, disease evolution, and possible treatments, described in the recent literature. Following infection, many patients reported a wide range of symptoms and complications, not self-limiting and independent from previous infection severity. SFN begins more frequently with distal limb burning pain and numbness, which accompany other dysautonomia, cognitive, visual, and osteoarticular disorders involving multiple organ systems. In an initial diagnostic suspicion, some tests might be useful as complementary examinations, such as nerve quantitative sensory testing, electromyography, and optic nerve tomography. Otherwise, definite diagnosis is reached with skin biopsy as the gold standard, along with corneal in vivo microscopy when ocular discomfort is present. Being a long-term condition, multiple and dissimilar symptomatic and disease-modifying drugs were employed for the treatment of this condition with the achievement of partial results, including steroids, pregabalin, gabapentin, duloxetine, vitamins, homotaurine and phosphatidylserine, alpha lipoic acid, immunosuppressants, and intravenous immunoglobulin therapy. PASC-SFN is a complex emerging disease and extremely challenging for physicians. At present, the only feasible management of PASC-SFN is represented by a multidisciplinary tailored approach, with future definitive protocols for diagnosis and treatment deemed essential.

## 1. Introduction

Somatosensory system alterations, often associated with neuropathic pain, chronic fatigue, and paresthesia and initially documented in fibromyalgia [1,2], rheumatoid arthritis [3,4], and connective tissue diseases [5,6], are considered relevant red flag symptoms of the post-acute sequelae of COVID-19 (PASC) syndrome.

Currently, after the emergency phase of the first and second waves of the pandemic, PASC represents a new intriguing “puzzle” of clinical manifestations with uncertain pathogenesis and unknown evolution which are estimated in about 10–30% of patients and might last, from infection, over 2 months and up to years [7,8,9]. The wide range of symptoms and complications of PASC involves multiple organ systems, including headache, osteoarticular symptoms, and cognitive and visual impairment, which necessitate a multidisciplinary approach [10,11].

In such a scenario, PASC-associated small fiber neuropathy (SFN) may be a rare post-infective complication of COVID-19 [12] that begins with allodynia in distal limbs [13,14] and might evolve into burning pain, numbness, thermal disarray, “stocking glove” sensation, and motor impairment [15].

SFN is a rare, disabling and not self-limiting disease of the peripheral nervous system that affects the small-diameter unmyelinated C-type fibers and/or thinly myelinated Aδ-type fibers which are present in the skin, muscles, nerves, and internal organs. SFN has uncertain pathogenesis and might have a kaleidoscopic onset and clinical presentation. For this reason, this new syndrome still does not have definitive classification criteria or diagnostic and therapeutic protocols and represents a challenge for physicians and specialists, being often underestimated or misdiagnosed as fibromyalgia or psychiatric and cognitive disorders [14,15].

In fact, the overlap with other comorbidities might hamper proper early clinical management, and the routine tests for sensitivity and thermal thresholds, such as neurophysiological assessments and electromyography (EMG), still present limits of specificity and sensitivity for the diagnosis of PASC-SFN.

Even though morphological alterations of small nerve fibers can be identified through a few newer techniques—such as in nerve ultrasonography or vivo corneal confocal microscopy (CCM) [3,16]—unfortunately, these methods are not yet widely adopted in routine clinical practice. At present, the histological analysis of skin biopsy remains the gold standard for the diagnosis of SFN [17,18]. However, the lack of standardized protocols and the limited availability of expertise in SFN-specific biopsy techniques are significant obstacles, often leading to delayed diagnosis.

A potential immune-mediated pathogenesis of SFN was hypothesized early on, with autoantibodies emerging as a promising area of research for classifying the disease [19,20]. Nevertheless, no definitive biomarkers have been identified so far [21] and the potential genetic predisposition to SFN remains unknown [22].

Furthermore, immunomodulator drugs, particularly intravenous immunoglobulins (IVIGs), were liberally considered in SFN treatment, although the lack of specific procedure rules might limit its “off label” use in many countries, requiring the overcoming of public institution or private assurance obstacles to proceed.

In the present systematic review, for the first time, we provide an overview of the diagnostic opportunities of PASC-SFN, its possible multiorgan involvement, clinical evolution, and the treatments employed so far. Moreover, we searched the literature for possible specific biomarkers that could serve to diagnose PASC-SFN early and, therefore, could be useful in future clinical protocols.

## 2. Materials and Methods

### 2.1. Search Strategy and Inclusion/Exclusion Criteria

The articles included in this systematic review, which followed the Preferred Reporting Items for Systematic Reviews and Meta-Analyses (PRISMA) 2020 guidelines, were selected by searching in PubMed (*n* = 59) and Embase (*n* = 63) for relevant literature without time limits, up to 25 July 2024, using the keywords [“small nerve neuropathy” AND “COVID-19”]. Subsequently, the search strategy, which was not registered in PROSPERO or in another relevant database, was refined to include relevant original studies and case series reporting PASC-SFN.

We selected titles reporting post-COVID-19 (PC) patients with clinical or histological diagnoses of SFN following COVID-19 infection and not present before. Cohorts derived from mixed populations of PC patients, other aspects of PASC, or also including COVID-19 vaccine reactions were also included. PASC patients were considered the population studied, while SFN was the index test/intervention. Clinical and diagnostic presentation, association with the new onset of other clinical manifestations, biomarkers, prognosis, and therapy were the outcomes of the review. In different studies, patients with PASC-SFN have been compared to healthy controls (HC) or patients with other asymptomatic PC conditions, PASC, or other neurological conditions such as postural orthostatic tachycardia syndrome (POTS). We included also cohorts without controls, otherwise the number of selected articles and investigated patients would have been limited.

**Figure 1:**
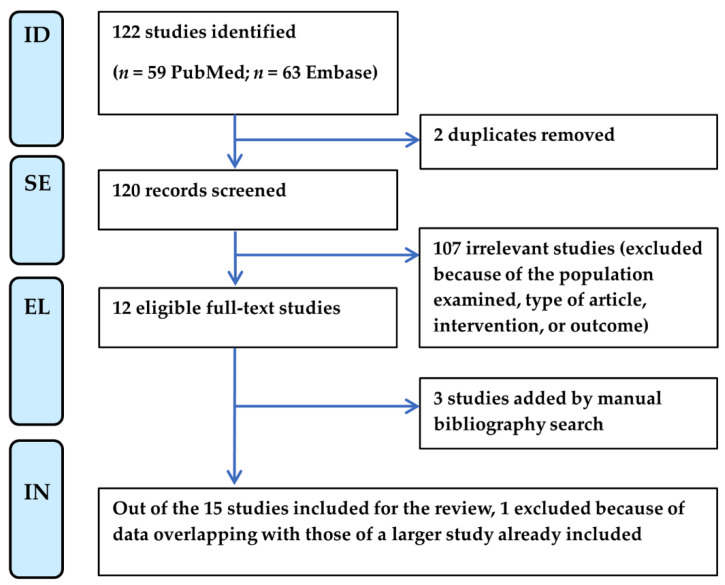
PRISMA flow chart of literature selection for systematic review. EL, eligibility; ID, identification; IN, inclusion; SE, selection.

Two independent reviewers (F.B. and M.M.) screened the searched titles and abstracts, assessed the full texts for eligibility, and extracted relevant data from qualifying studies according to the flow chart shown in Figure 1. Any discrepancy at each stage was resolved through discussion moderated by a third reviewer (M.D.C.). This process adhered to the protocol schedule, established from 26 July 2024 to 30 September 2024, following the PRISMA checklist (Supplementary Table S1).

We excluded studies based on the population examined (i.e., studies that enrolled patients with neurological symptoms present before COVID-19 infection, patients with a previous diagnosis of fibromyalgia, or the same patient cohort of studies already included). Moreover, we excluded published conference abstracts, reviews, commentaries, book chapters, and editorials. Relevant letters and case series with detailed methodologies and results were instead considered, if they provided prevalence data.

Eligible studies were also found manually from the bibliographies of reviews and articles, published in the timeline of the review, that cited the articles initially selected for review (“snowballing”) [23].

### 2.2. Literature Data Collection

We collected data regarding the demographic (i.e., sex ratio and mean or median age), COVID-19 severity, and SFN (i.e., clinical attributes, time from infection, acute or subacute onset, and symptoms) of patients, possible association with other PASC manifestations (in particular, dysautonomia and rheumatic, ophthalmic, and cognitive disorders), and follow-up and treatments, all expressed as percentages.

Based on the information contained in the articles, we classified COVID-19 severity according to the World Health Organization (WHO) guidelines (0–10), and PASC according to the WHO definition of long COVID (i.e., onset of symptoms within 90 days of the first day of COVID symptoms that did not last for >2 months) [7,8].

We searched the selected papers for the six main clinical SFN symptoms as follows: fatigue, motor impairment, burning pain, numbness, thermal disarray, and stocking glove [14,17].

We considered clinical neurological parameters for SFN definition, diagnostic protocol if present, histological criteria and technique, as well as other possible instrumental findings (i.e., neurological, ocular, and rheumatological).

Patients with clinical diagnoses without histological evidence of SFN were also included in the study.

We collected also data on autoimmunity, follow-up, persistence or progression of neurological symptoms, and possible treatment outcomes.

## 3. Results

### 3.1. PRISMA Literature Selection

The PRISMA flow chart of the systematic literature review selection is shown in Figure 1. Starting from a total of 122 publications from PubMed and Embase, most of the articles were excluded because of the type of article (i.e., abstract, editorial, book chapter, or review) and intervention. Only one article was excluded due to the type of patient population investigated as the diagnosis of SFN was performed before COVID-19 onset [24]. Next, three papers were added by manual search [13,14,16] because they cited the articles initially selected. Of the fifteen studies selected, we finally eliminated another one because it reported data from a cohort of patients partly overlapping with those of another larger study already included [21,25].

Since some studies included mixed populations with patients with SFN successive to COVID-19 vaccination [14,26], whenever possible we selected data about only PASC-SFN, as shown in Table 1 [13,14,15,16,21,26,27,28,29,30,31,32,33,34]. Only one study included one patient with post-vaccine SFN that we did not show in the results [26].

Only nine papers (excluding case series) were assessed for risk of bias using an adapted version of the modified-Newcastle–Ottawa Scale (m-NOS) for case–control studies (Supplementary Table S2) based on selection (i.e., disease definition and representativeness; score 0–4), comparability (0–1), and ascertainment of SFN (records 0–1, same method 0–1, non-response rate 0–1 = 0–3).

Even if the studies included showed a good quality, because of the limited number of patients examined and the great variability of methods employed in the items selected that included also case series, we decided a priori to not perform a meta-analysis of the data. For the same reason, we have not performed a specific analysis of sensitivity.

All papers included were monocentric studies. Moreover, almost all the studies included were retrospective and cross-sectional, except for one prospective study [13] and a few case series [26,27,28]. The studies reported traditional clinical evaluations and did not employ telemedicine. Patients were compared to HCs [16,21,29,30,31,32], PASC-SFN treated with IVIG (vs naïve) [15], PASC painful and PC non-painful patients [14,15,21], and patients with postural orthostatic tachycardia syndrome (POTS) [21] (Table 1).

### 3.2. Clinical Characteristics and Diagnosis of PASC-SFN

The characteristics of patients with an SFN onset after COVID-19 are shown in Table 1. The patients displayed a mean age ranging from 34 to 57 years old, with a prevalence of the female sex in 11 out of 14 studies (Table 1).

PASC began 4 to 19 days after COVID-19 infection, with acute onset. The previous COVID-19 infection was mild (WHO 2) in most of the studies (Table 1). Falco et al. reported that the severity and duration of disease was independent of PASC-SFN manifestations [13]. Moreover, Bandinelli et al. demonstrated that both the residual interstitial lung involvement and the time between a COVID-19 negative swab and PASC onset were similar between PASC-SNF and PASC patients [14].

When neurological symptoms were described in SFN patients, they were referred to as burning pain and numbness in most of the studies (Table 1). Though not always described in all issues, other clinical aspects present in a high percentage of cases included fatigue [13,14,27], motor impairment [14,27,28,33], thermal disarray [13,14], and stocking glove [13,14,34].

Neuropathic pain affecting patients with PASC-SFN was studied through routinary clinical electrodiagnostic and autonomic function tests [33], or with pre-defined clinical criteria [13,14,21,29,30] (Table 1). Azcue et al. [29] used TSA-2 quantitative sensory testing (QST) to measure the response of C and Aδ small fibers to pain and temperature and concluded that the ability to detect heat was lower in PASC patients than in HCs (Table 1).

In particular, Falco et al. [13] followed the Besta criteria [17] that consider the diagnosis of SFN relying on the presence of a preserved nerve conduction study alongside distally distributed sensory signs, which included diminished thermal pain sensation, hyperalgesia, and/or allodynia, at bedside clinical examination, complemented by at least one of the following confirmatory tests: (1) abnormal cold and/or warm detection threshold, as determined by QST, and (2) a reduction in intraepidermal nerve fiber density (IENF/mm) in skin biopsy samples from the distal calf.

Even if, to date, skin histology represents the gold standard for the diagnosis of SFN, out of 14 studies selected only 7 reported histological criteria for SFN, and their sensitivity or specificity was not precisely studied in these PASC-SFN patients. Of note, in the studies of Falco et al. [13] and Oaklander et al. [33], patients with clinical features highly suggestive of SFN but without biopsy criteria were also considered for statistical analysis.

**Figure 2:**
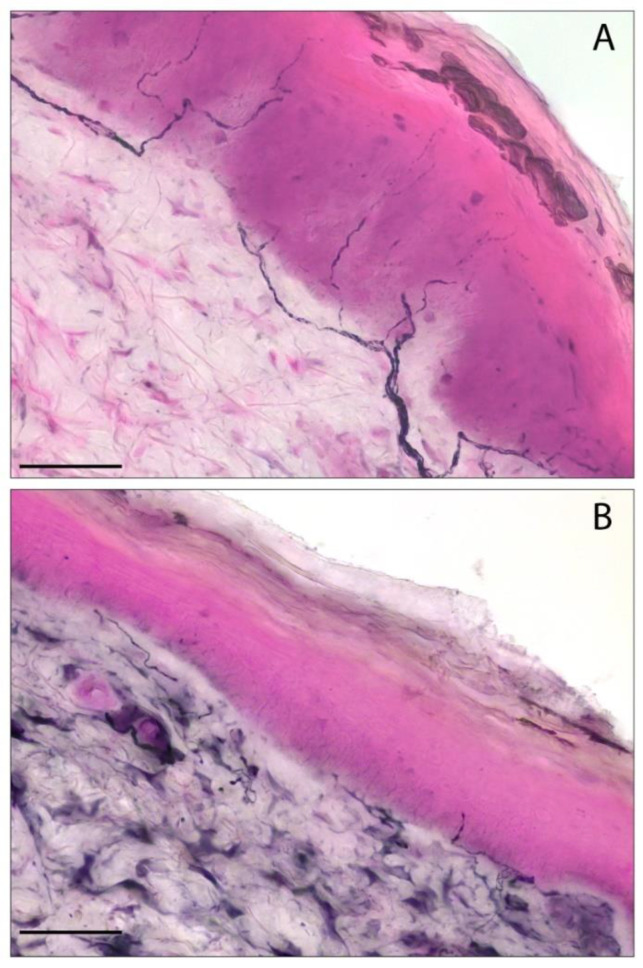
The detection of intraepidermal nerve fibers in skin biopsies by PGP9.5 immunohistochemistry. (**A**) A representative image of a skin biopsy sample with a normal distribution of epidermal nerve fibers. (**B**) The reduction in intraepidermal nerve fiber density (IENF/mm) in a follow-up patient with a final diagnosis of small fiber neuropathy. Scale bar: 50 µm. The staining was performed using an immunohistochemical approach with a primary antibody specific to PGP9.5, followed by a secondary antibody conjugated to horseradish peroxidase, and visualized with a blue chromogen/peroxidase substrate. The images are from a PASC patient with a first negative biopsy (**A**) which evolved to a positive one after six months of clinical follow-up (**B**) who was enrolled in our recent study [14]. PASC: post-acute sequelae of COVID-19.

Four articles used the same histological skin tissue biopsy protocol described by Lauria et al. [13,14,18,21,34]: two punch skin biopsies from the distal leg and proximal thigh were immunoassayed using an anti-PGP9.5 antibody to evaluate IENF/mm (Figure 2). Oaklander et al. did not describe their upper thigh and lower leg skin biopsy protocol [33], while in another study [15] a punch biopsy performed unilaterally on the foot and thigh was investigated by bright-field immunohistochemistry to assess IENF/mm in SFN patients, without describing the protocol in detail.

In the case report of Panagiotides et al. [27], a biopsy of the sural nerve, which contains both myelinated and unmyelinated sensitive fascicles, was used for SFN diagnosis because of its good accessibility. After extraction, the tissue was used for light microscopy, by employing standard histological stains and immunohistochemistry, and for electron microscopy, as well as for protein, DNA, and RNA molecular analyses.

Only in the study of Bandinelli et al. [14] was IENF/mm compared between patients with PASC-SFN (a small population mixed with post-COVID-19-vaccine SFN patients) and PASC with similar age and sex distributions, highlighting significant differences.

Furthermore, three studies assessed nerves in the central and paracentral cornea with in vivo CCM [16,30,32] in order to acquire the characteristics of the corneal epithelium and sub-basal nerve fiber plexus up to the anterior, middle, and posterior stroma. Seven parameters were considered [35] as indicator of the structural complexity of the corneal nerve: (1) corneal nerve fiber density, i.e., the total number of nerves/mm^2^ [16,30,32]; (2) corneal nerve branch density (CNBD), i.e., the number of second-order branches emanating from primary axons/mm^2^ [16,30,32]; (3) corneal nerve fiber length, i.e., the total length of all nerve fibers and branches (mm/mm^2^) [16,30,32]; (4) corneal nerve total branch density, i.e., the total number of branches/mm^2^ [30,32]; (5) corneal nerve fiber area, i.e., the total nerve fiber area (mm^2^/mm^2^) [30,32]; (6) corneal nerve fiber width, i.e., the average nerve fiber width (mm/mm^2^) [30,32]; and (7) corneal nerve fractal dimension [30,32]. The cell count of the images was also calculated in order to quantify the incidence of neuromas (total number of neuromas/mm^2^) [32], including beaded axons (total number of beaded axons/mm^2^) [30,32], fiber tortuosity [32], and the density of dendritic cells (DCD) in the center of the cornea (total number of dendritic cells/mm^2^) [16,30,32]. CNBD was lower in patients with SFN and PASC than HCs in all three studies [16,30,32] (Table 1), while DCD was higher in patients with SFN and PASC with neurological symptoms only in two studies [16,32]. Furthermore, Bandinelli et al. reported a reduction in the peripapillary retinal fiber nerve layer through ocular computerized tomography (OCT) of the optic nerve in 50% of PASC-SFN patients [14].

### 3.3. Accompanying Disorders in PASC-SFN Patients

“Visual fog” and ocular discomfort, described in 4 out of 14 studies [14,27,30,32], were frequent accompanying symptoms in PASC-SFN patients, with visual impairment described in 3/14 [14,27,30], dry eye in 1/14 [30], and pain, redness, and burning eye in 1/14 [32], as shown in Table 1.

In addition, arthritis and arthralgia were described in 4/14 studies [14,27,32,34]. A possible evolution of such a condition into established arthritis was shown only in one issue study [14].

Dysautonomia was reported in 5/14 studies. In particular, POTS was described in almost 30% of patients [15,27,34]. Furthermore, Novak et al. showed, in tilt tests, a higher heart rate response with lower end-tidal CO_2_, and, in cerebral Doppler tests, a lower blood flow in PASC-SFN patients compared to HCs [21].

Finally, the cognitive and memory impairment (also called “brain fog”), reported in 3 out of 14 studies [13,14,32], ranged from 100% [14] to 32.5%of patients [32]. Depressive [13] and language [32] disorders were also described. Rarer accompanying symptoms included anosmia, tinnitus, and dizziness (Table 1).

**Figure 3:**
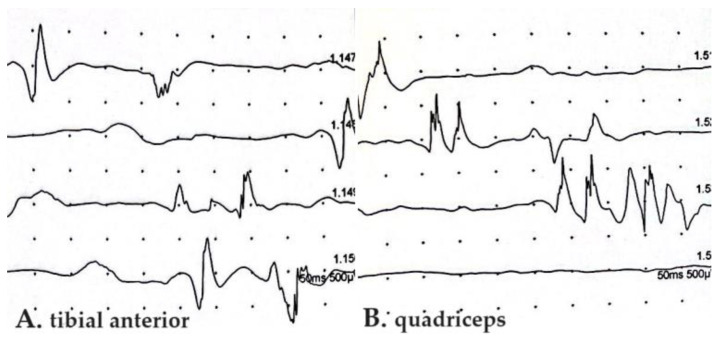
Electromyography of tibial anterior (**A**) and quadriceps (**B**) muscles in PASC-SNF patient enrolled in our recent study [14]. Abnormalities of motor unit action potentials with decrease in amplitude and polymorphic aspect are shown. SFN, small fiber neuropathy; PASC, post-acute sequelae of COVID-19.

Motor unit action potential (MUAP) abnormalities in EMGs accompanying SFN were described only by Bandinelli et al., showing a short duration, small amplitude, and polyphasic aspect, without the spontaneous activity of positive sharp waves and fibrillations [14] (Figure 3).

No specific dermatosis as an accompanying disorder was described in the studies analyzed.

### 3.4. Biomarkers for PASC-SFN

C-reactive protein (CRP) seemed more elevated in PASC-SFN patients than in PC painful patients without SFN [14], a finding confirmed in the report by McAlpine et al. showing an increase in CRP in moderate and severe SFN patients [15].

Autoantibodies were evaluated only in 5/14 studies, with non-univocal methods [14,15,27,33,34]. Only McAlpine et al. used trisulfated heparin disaccharide/fibroblast growth factor receptor 3 (TS-HDS/FGFR3) antibodies as specific markers for SFN [15]. In the aforementioned study, out of ten PASC-SFN patients three were positive for TS-HDS IgM and three for FGFR3 IgG. As far as the other articles are concerned, while two studies considered antinuclear antibody (ANA) positivity an exclusion parameter [33,34], others described ANA/ENA [14,27] or perinuclear anti-neutrophil cytoplasmic antibody (p-ANCA) positivity [27] in PASC-SFN patients. In particular, Bandinelli et al. [14] found a mildly higher prevalence of ANA in patients with PASC-SFN compared to those with PASC (87.5% vs. 50%, respectively), while anti-spike protein and Anti-SARS-CoV-2 antibody levels were not different between the two groups.

### 3.5. Evolution and Possible Treatments of PASC-SFN

As shown in Table 2, in all 8 out of 14 studies that investigated the possible evolution of PASC-SFN, SFN symptoms seemed persistent during the time and not self-limiting [13,14,15,26,27,28,31,33,34].

In the study of Abrams et al., duloxetine, pregabalin, and gabapentin treatments showed controversial results in PASC-SFN patients (*n* = 6) and seemed more effective in PASC patients (*n* = 7) with normal skin biopsies [34].

Bandinelli et al. showed that PASC-SFN maintained a stable IENF/mm at histological follow-up, after almost six months, independently of changes in symptoms. Furthermore, in PASC-negative patients at first skin biopsy with persistent symptoms suggestive of SFN, a second histological examination after six months showed a progressive decrease in IENF/mm [14] (Figure 2).

Pregabalin, gabapentin, and duloxetine were used in almost all studies without the complete resolution of symptoms (Table 2).

While Bandinelli et al. reported, at 12 months follow up, the efficacy of corticosteroids, hydroxychloroquine, multiple integrators for the nervous system (i.e., homotaurine and phosphatidylserine, folate, and alpha lipoic acid), and other immunosuppressants (i.e., SLZ, methotrexate, and mycophenolate) [14], the case report by Panagiotides et al. showed that IVIG seemed more effective than other immunomodulatory treatments (i.e., steroids, hydroxychloroquine, and rituximab) [27]. Indeed, IVIG led to a higher percentage of improvement [15,27,33] or resolution of symptoms after six months of treatment [15], but with possible relapse upon the weaning of treatment [15,27].

Despite treatments, in all cases, the prognosis of SFN seemed controversial and did not lead to a complete resolution.

## 4. Discussion

Here, we report the findings of a systematic review of a few reports highlighting that PASC-SFN is a not-self-limiting and invaliding disease, diagnosed and treated with protocols not largely diffused and currently not completely validated, and associated with heterogeneous multiorgan manifestations.

For this reason, the management and care of this new syndrome requires a multidisciplinary approach and long-term management that are still not well defined [36].

The strength of this systematic review lies in its ability to describe this complex condition, providing up-to-date information on diagnoses and current management recommendations from the literature, with a focus on specialists, as well as to shed light on possible future perspectives for the diagnosis and treatment of PASC-SFN. However, current limitations include the paucity of data regarding the prevalence and duration of PASC-SFN, as well as the absence of unified classification criteria and standardized protocols.

The diagnosis of SFN can be suspected based on the presence of characteristic symptoms specifically localized in distal limbs and arms, even if clinical evaluations appeared largely concentrated only on burning pain and numbness symptoms that are common to other neuropathic conditions. Moreover, routine clinical diagnostic tests seemed inconclusive, except for a little evidence on quantitative sensory testing (QST) for heat sensitivity [29] that was also included in one of the few protocols used, known as the Besta criteria [13,17].

PASC-SFN is often associated with autonomous nervous system failure that could be investigated with nonroutine diagnostic procedures, such as a tilt test with the observation of hearth rate increase, hypocapnia, and the reduction in cerebral blood flow [21].

Recently, two studies, using in vivo CCM, revealed a decrease in corneal nerve branch density in PASC patients, but further studies might more deeply evaluate any other divergent parameters shown in these issues (Table 1) [30,32]. Moreover, the retinal nerve fiber layer thickness reduction in the optic nerve was described previously by only one study in PASC-SFN [14,35].

EMG was used prevalently for large fiber neuropathy exclusion and might reveal, in PASC-SFN, only accompanying MUAP abnormalities, described as having a short duration, small amplitude, and polyphasic aspects, as previously demonstrated frequently in PC patients [37] and in idiopathic SFN [17,38].

Finally, only four articles [13,14,21,34] used Lauria’s immunohistochemical protocol for the analysis of skin biopsies [18], which currently is considered the gold standard for the assessment of intraepidermal nerve fiber density at the lower limbs.

Nevertheless, even though over the last few decades skin biopsy has contributed to demonstrating small fiber involvement in an increasing number of pathologies, such as diabetes, fibromyalgia and other rheumatic diseases and post-infective syndromes (HIV, C and B hepatitis, and leprosy), studies addressing its sensitivity and specificity to different etiologies are missing [39], as is also shown in our review.

Furthermore, it is interesting to note that repeated skin biopsy over time was previously used to assess the efficacy of pharmacological and non-pharmacological treatments [39], but only Bandinelli et al. employed this approach to follow-up PASC patients, showing its possible usefulness in the revaluation of patients who were initially classified as negative and subsequently evolved into positive cases. From this perspective, future multicenter studies might widely evaluate the potential of such an approach for case follow-up in larger cohorts.

As we summarized in this review, until now PASC-SFN has been rarely described in the literature, and a previous review on skin biopsies focused only on dermatosis in the course of COVID-19 infection, without addressing specific nerve abnormalities [40]. In the future, if skin biopsy becomes more widely adopted, the understanding and recognition of SFN prevalence among long-COVID-19 patients might significantly improve.

Amongst the possible biomarkers, autoantibodies seemed to be the most intriguing area of interest [19] and specific markers like TS-HDS/FGFR3 antibodies might be present in SFN patients with autonomic dysfunction [41], even if their diagnostic value is still uncertain in the literature [15,41,42].

In PASC-SFN, specific biomarkers were rarely investigated or were not specific, except for McAlpine et al. who found in almost 30% of TS-HDS/FGFR-3 autoantibodies patients [15], although their exact pathophysiological role in the peripheral nervous system has not been definitely demonstrated.

Though a high prevalence of ANA in PASC-SFN [14] and an efficacy of immunosuppressive [14] and IVIG treatment [14,15,27,33] have been reported, considering the small number of patients investigated until now, a definitive demonstration of the implication of autoimmune pathogenic mechanisms is still lacking.

Given the lack of a diffuse specific algorithm for this rare and emerging disease, the treatment strategy is often off-label and exploratory, and might be improved with future larger cohort studies. Indeed, despite an initial enthusiasm for the use of IVIG in the treatment of SFN [19,42], the benefits of this therapy are still uncertain because of the potential for relapse upon short term withdrawal, which indicates the need for further trials to determine its real efficacy in the future [43]. The current support treatment for SFN consists of different classes of neuropathic pain medication, including anticonvulsants, antidepressants, opioids, and topical agents, which seem to provide mild palliative control for burning pain but less so for the other symptoms; only steroid therapy, commonly employed in patients with acute SFN, might present a high risk of recurrence after withdrawal [43], as shown by Panagiotides et al. [27].

Immunomodulators and immunosuppressive drugs (e.g., hydroxychloroquine, sulfasalazine, methotrexate, and mycophenolate) have been employed with controversial results [14,27]. For instance, Panagiotides et al. reported only one case and did not specify the duration of follow-up [27]. Additionally, Bandinelli et al. demonstrated the efficacy of a combination therapy involving vitamin B6, B12, D3, folate, homotaurine, phosphatidylserine, and alpha lipoic acid for long-term use in PASC-SFN, particularly in addressing brain fog. However, this finding lacks broader support in the literature [14].

A significant limitation of the studies is the maximum follow-up period of 12 months [14]. It is reasonable to hypothesize that longer observation periods are necessary to fully evaluate therapeutic outcomes, particularly in less-explored aspects such as fatigue, motor impairment, and thermal regulation abnormalities.

## 5. Conclusions and Clinical Implications

PASC-SFN is an invaliding and non-self-limiting condition which is one of the more severe and long-term sequalae of PASC.

In this systematic review, for the first time, we summarized the current knowledge about clinical and treatment approaches to PASC-SFN and possible biomarkers for this condition.

The recent literature supports persistent inflammation and immune activation as plausible mechanisms, but at present it remains unclear whether they are solely responsible for PASC-SFN. We recognize the uncertainty of the surrounding pathogenetic mechanisms, and we might encourage future experimental studies that can validate these hypotheses, such as longitudinal analyses of cytokines and autoimmunity in patients with PASC-SFN.

Even if thermal disarray and distal limb localization seemed to be typical aspects of the disease, the clinical approach is probably too concentrated on burning pain and numbness, which are symptoms that are difficult to distinguish from other neuropathic conditions.

In the future, flow charts and protocols are deemed essential to create red flags to send to second/third-level neurological and rheumatological evaluations, with possible successive skin biopsy proving diagnosis. In fact, even if skin biopsy is considered the gold standard for a definitive diagnosis of SFN, at present its specificity for PASC-SFN is not definitely demonstrated, as in other post-infective conditions, and its use is not largely diffuse in outpatient clinics.

Furthermore, EMG seemed to have more limited but interesting aspects highlighting MUAP abnormalities that should be studied more deeply in the future.

In addition, systemic symptoms may accompany PASC-SFN and require a multi-faceted approach involving multiple specialists, which highlights the need to implement future effective diagnostic protocols. In particular, modern corneal and retinal examination techniques seem to be very promising for the comprehension of “visual fog” in PASC-SFN diagnosis, and they could be further developed in future research.

An increase in autoantibodies was sporadically reported, but not diffusely studied. Therefore, the possible autoimmune component of PASC-SFN remains to be clarified. Indeed, the identification and validation of specific biomarkers, such as autoantibodies, will require studies involving larger cohorts of patients.

Finally, it should be underlined that the imaging and laboratory techniques employed to date for the early diagnosis of PASC-SFN were almost pioneering and, since they have been applied to small cohorts, a definitive demonstration of their sensitivity and specificity will be required. Concerning treatments, despite some promising results, the efficacy of IVIG and immunosuppressants remains uncertain, and further studies on larger cohorts will be essential to understand their long-term outcome.

## Figures and Tables

**Table 1:** The main patient characteristics and results of the 14 studies included in the systematic review.

Study, Year of Publication, Country [ref.]	Sample Size	Age at Diagnosis (Years)	SFN Histologic (H) or Clinical (C) Criteria	Comparison to Controls	WHO COVID-19 Severity (*n* Patients)	SFN Onset Since Infection (Days)	SFN Symptoms and Localization	PASC Symptoms and Biomarkers
Abrams et al., 2022 USA [34]	6 PASC-SFN (6 females); 5 PASC	48 (8.4) mean	Lauria (H) criteria; NA	Distal-reduced pinprick in PASC-SFN vs. normal in PASC	2 (4), 5 (1), 6 (1)	10 (7), mean (SD)	66.6% BP; 100% N; 33% SG; 100% lower limbs; 33.3% also upper limbs	16.6% arthralgia; 33% POTS; 16.6% tachycardia; 100% ANA negative
Azcue et al., 2023 Spain [29]	87 PASC (17/87 PASC-SFN; 62 females, 25 males); 50 HCs and 50 with chronic fatigue syndrome	NA	SFN clinical screening list; Sudoscan and QST	SFN screening list higher in PASC-SFN (*p* = 0.001); Sudoscan NS; QST for heat lower than HCs (*p* = 0.001)	NA	NA	NA	NA
Bandinelli et al., 2024 Italy [14]	7 PASC-SFN and 1 PCV-SFN (5 females, 3 males); 15 PASC-PCV	57 (14) mean	Lauria (H) and Bradford Hill criteria (C)	Small nerve density/mm at biopsy (PASC-PCV-SFN) lower (*p* = 0.003); OCT abnormalities higher than PASC-PCV (*p* = 0.01)	2 (6), 5 (1)	4 (1–11), median (IQR)	100% BP, N, F, MI, TD and SG; lower limbs	Arthritis (100%); abnormal ocular OCT (6.5%); brain and visual fog (100%); EMG-MUAPs (75%); HRCT (62.5%); DLCO abnormalities (37.5%); ANA+ (87%)
Barros et al., 2022 Spain [30]	23 PASC (21/23 PASC-SFN; 18 females, 5 males); 46 HCs	NA	In vivo corneal microscopy	CNBD and CNFL lower (*p* < 0.01 and *p* < 0.05) and DCD higher (*p* = 0.05) in PASC vs. HCs	2 (22), 5 (1)	NA	NA	100% corneal symptoms; 34% Surface Disease Index test severe; 17.3% Schirmer test positive;
Burakgazi et al., 2022 EUR [31]	2 SFN (2 females)	55.5 (9.1) mean	NA	NA	2 (2)	21 (9.8), mean (SD)	100% BP, N, TD; upper and lower limbs	NA
Bitirgen et al., 2022 Turkey [16]	22 neurological PASC (10 females, 12 males); 18 PC; 30 HC	48.3 (13.1) mean	NA	CNBD (*p* = 0.02), CNFL (*p* = 0.01), CFND (*p* = 0.03) lower than HCs; CFNL (*p* = 0.02) and CFND (*p* = 0.04) lower than PC; DCD higher than HCs (*p* = 0.001)	2 (22), 4 (11), 5 (4), 7 (3)	26.6 (9.1), mean (SD)	NA	NICE-Q neurological score (0–7) at 12 weeks: 1.0 (0.5–2.0), median (IQR)
Falco et al., 2024 Italy [13]	12 PASC-SFN (7 females, 5 males); 14 painful PASC	49.9 (11.7) mean	Besta and Lauria (H) criteria (only 6 positive at biopsy)	Higher sensory loss and mechanical hyperalgesia in PASC-SFN vs. PASC (*p* = 0.0026); smaller distal area involved (*p* < 0.001)	2 (7), 4 (2), 5–6 (3)	17.3 (20.3), mean (SD)	100% BP and SG; 83% F; 57.7% TD; upper and lower limbs	83% depression and cognitive impairment
McAlpine et al., 2024 USA [15]	9 PASC-SFN treated with IVIG (5 females, 2 males); 7 untreated PASC-SFN	48 (40–62) median	WHO (C) definition of PASC; skin biopsy (H)	Clinical response to IVIG (*p* = 0.0001)	2 (5), 4–5 (4)	14 (14–21), median (IQR)	100% BP and N; NA	Dysautonomia (89% IVIG, 86% untreated); 33% TS-HDS/FGFR3+
Midena et al., 2022 Italy [32]	151 PASC (68 females, 83 males); 46 HCs	56.8 (14.2) Mean	In vivo corneal microscopy	CNBD, CTBD, and CNFW reduced vs. HCs (*p* = 0.01, *p* = 0.05, and *p* = 0.005); NBe and DCD higher than HCs (*p* = 0.004 and *p* = 0.0001)	4 (4), 5 (90), 6 (30),7 (7), 8 (16)	19.3 (10.5), mean (SD)	NA	Arthralgia (45%); visual (23.2%); cognitive (32.5%); syncope (11.9%)
Novak et al., 2022 USA [21]	15 PASC, (10/15, PASC-SFN; 12 females, 3 males); 15 POTS (14/15 with SFN); 11 HCs	35.8 (7.9) Mean	Brigham (C) and Lauria (H) criteria	In PASC and POTS autonomic failure (*p* < 0.005), increased heart rate (tilt test), decreased cerebral blood flow (*p* < 0.001), reduction in end-tidal CO_2_, (*p* < 0.001)	NA	NA	NA	In PASC, 100% autonomic failure and reduced orthostatic cerebral blood Doppler flow; 87% hypocapnia
Oaklander et al., 2022 USA [33]	17 PASC-SFN (10/16 positive at biopsy; 11 females, 6 males)	43 (21) mean	Electrodiagnostic and autonomic tests; skin biopsy (H)	NA	2 (17), 6 (1)	6 (1.3), mean (SEM)	11.7% MI; 100% BP and N; lower limbs	ANA negative
Panagiotides et al., 2023 Austria [27]	1 PASC-SFN (female)	34	Sural nerve biopsy (H)	NA	NA	Same month of infection	F, BP, N, MI; acute; upper and lower limbs	Arthritis; visual impairment; POTS; p-ANCA and anti-Scl34+
Shouman et al., 2021 USA [28]	1 PC (male)	52	NA	NA	NA	NA	50% MI; 100% BP and N; upper and lower limbs	NA
Schwartz et al., 2023 USA [26]	1 PC SFN (female)	54	NA	NA	2 (1)	NA	BP and N; lower limbs	NA

Data expressed as mean and standard deviation (SD) or SEM (standard error mean), or median and interquartile range (IQR). WHO scale for clinical progression of COVID-19 (0–10): mild (1–3); moderate (4–5); severe (6–9); dead (10). ANA, antinuclear antibodies; BP, burning pain; CNBD, corneal nerve branch density; CNFL, corneal nerve fiber length; CNFW, corneal nerve fiber width; CTBD, corneal total branch density; DCD, density of corneal dendritic cells; DLCO, carbon monoxide lung diffusion test; F, fatigue; HCs, healthy controls; HRCT, high-resolution CT of the chest; IVIG, intravenous immunoglobulins; MI, motor impairment; NA, not applicable; N, numbness; NBe, number of corneal beaded axons; p-ANCA, perinuclear anti-neutrophil cytoplasmic antibody; PASC, post-acute sequelae of COVID-19; PC, post-COVID-19; PCV, post-COVID-19 vaccine; POTS, postural orthostatic tachycardia syndrome; QST, quantitative sensory testing; SFN, small nerve neuropathy; SG, stocking glove; TD, thermal disarray; SFNSL, small fiber neuropathy screening list; TS-HDS/FGFR3, trisulfated heparin disaccharide/fibroblast growth factor receptor 3 antibodies.

**Table 2:** Studies that reported treatment for PASC-SFN, outcome and prognosis at follow-up.

Authors, Year of Publication, Country [ref]	Duration of Follow-Up of PASC-SFN	Treatment	Outcome	Prognosis
Abrams et al., 2022 USA [34]	8–12 months	Gabapentin, duloxetine, amitryptline	4 improved; 2 poorly controlled	+/−
Bandinelli et al., 2024 Italy [14]	12 months	Steroids, vitamin B6-B12-D3, homotaurine and phosphatidylserine, folate, alpha lipoic acid, pregabalin, HCQ, MTX, SLZ, mycophenolate	Improvement of brain fog, F, MI, BP, N, TD, SG at 12 months; no improvement of visual fog	+/−
Burakgazi et al., 2022 USA [31]	6–8 months	Pregabalin, duloxetine, amytriptiline	Improvement of BP only in 50%; no improvement of N	−
Falco et al., 2024 Italy [13]	NA	Gabapentin, pregabalin, duloxetine	NA	NA
McAlpine et al., 2024 USA [15]	6 and later (not specified) months	Patients previously treated with gabapentin, pregabalin, and/or duloxetine without resolution, candidates for IVIG	33% improvement and 66% resolution after 6 months; 55.5% relapse upon weaning of treatment, later	+/−
Oaklander et al., 2022 USA [33]	4 weeks	Corticosteroids in 35.3% (6/17) and IVIG in 35.3% (6/17)	88.2% improvement; 11.7% unvaried; 75% of IVIG patients ameliorated > 40%	+/−
Panagiotides et al., 2023, Austria [27]	NA	Gabapentin, high dose of corticosteroids, IVIG, hydroxyclochine, rituximab	Low improvement only after high dosage of steroids and IVIG in BP, but not F, MI, or N	−
Shouman et al., 2021, USA [28]	NA	Gabapentin and topical lidocaine cream	Improvement of BP	+
Schwartz et al., 2023, USA [26]	NA	Amitriptyline, gabapentin, pregabalin, or capsaicin	Not responsive	−

SFN, small fiber neuropathy; PASC, post-acute sequelae of COVID-19; BP, burning pain; F, fatigue; HCQ, hydroxychloroquine; IVIG, endovenous immunoglobulins; MI, motor impairment; MTX, methotrexate; N, numbness; SLZ; sulphasalazine; SG, stocking glove; TD, thermal disarray; NA, not available.

## Data Availability

No new data were created or analyzed in this study. Data and protocol are available if requested.

## Supplementary Materials

The following supporting information can be downloaded at: https://www.mdpi.com/article/10.3390/microorganisms13020328/s1, Table S1: The checklist of the systematic review according to Page MJ, McKenzie JE, Bossuyt PM, Boutron I, Hoffmann TC, Mulrow CD et al., The PRISMA 2020 statement: an updated guideline for reporting systematic reviews. BMJ 2021;372:n71. doi: 10.1136/bmj.n71; Table S2: Quality assessment of Modified Newcastle–Ottawa scale (m-NOS) was performed in 9 studies, excluding papers presenting only case series to which it could not be applied.
